# High Prevalence of Lesions of Systemic Hypertension in Bile-Extracted Asiatic Black Bears (*Ursus thibetanus*) and Associated Renal Disease

**DOI:** 10.3390/ani15131940

**Published:** 2025-07-01

**Authors:** Monica K. H. Bando, O. Lynne Nelson, Kyle Taylor, Rance Sellon, Clark Kogan, Jill Robinson, Emily Drayton, Claudia Hartley, David Donaldson, Chris Linney, Hannah Stephenson

**Affiliations:** 1 Veterinary Clinical Sciences, College of Veterinary Medicine, Washington State University, 100 Grimes Way, ADBF, P.O. Box 646610, Pullman, WA 99164, USA; olnelson@wsu.edu (O.L.N.); rsellon@wsu.edu (R.S.); 2 Veterinary Microbiology and Pathology and Washington Animal Disease Diagnostic Laboratory, College of Veterinary Medicine, Washington State University, P.O. Box 647040, Pullman, WA 99164, USA; k.taylor@wsu.edu; 3 Center for Interdisciplinary Statistical Education and Research, Washington State University, Abelson Suite 227, Pullman, WA 99164, USA; clark.kogan@wsu.edu; 4 Animals Asia Foundation, Room 1501, Tung Hip Commercial Building, 244-252 Des Voeux Road, Central, Sheung Wan, Hong Kong; jrobinson@animalsasia.org (J.R.); edra4815@gmail.com (E.D.); 5 Langford Veterinary Services, University of Bristol Veterinary School, Langford, Bristol BS40 5DU, UK; claudia.hartley@ed.ac.uk (C.H.); d.donaldson@ed.ac.uk (D.D.); 6 Willows Veterinary Centre & Referral Service, Highlands Rd, Shirley B90 4NH, UK; chris.linney@paragonreferrals.co.uk; 7 HS Cardiology Ltd., Dalton House, 9 Dalton Square, Lancaster LA1 1WD, UK; hannah@hscardiology.co.uk

**Keywords:** aortic aneurysm, Asiatic black bear, bear bile, hypertensive retinopathy, left ventricular hypertrophy, renal disease, systemic hypertension, *Ursus thibetanus*

## Abstract

Approximately 17,000 bears undergo bile extraction in facilities across Asia for traditional medicines despite the availability of proven alternatives. Bears are confined to cages and bile harvested from the gallbladder via needle aspiration, implanted catheters, or transabdominal fistulas. Bile-extracted bears develop numerous detrimental conditions, including abnormal repetitive behaviors, emaciation, dental disease, cholecystitis, hernias, abscesses, and neoplasia. A high prevalence of aortic dilation, commonly seen with systemic hypertension, was reported in bile-extracted bears, and aortic aneurysm rupture/dissection was the third leading cause of death in a population of 600 formerly bile-extracted bears. A high incidence of renal disease, a common cause of systemic hypertension in other species, was also identified in this population. We hypothesized that renal disease was positively correlated with lesions of systemic hypertension in bile-extracted bears. Archived medical records, imaging, and samples from 180 formerly bile-extracted bears were analyzed. Hypertensive retinopathy, left ventricular hypertrophy, and aortic dilation were used as validated correlates of systemic hypertension. The majority (76.1%) of bears exhibited at least one systemic hypertension lesion, and 62.8% had two or more lesions. Left ventricular hypertrophy was most common, followed by aortic dilation/aneurysm. Lesions of systemic hypertension were positively correlated to renal disease parameters of serum creatinine and renal histopathology. Understanding the etiology of systemic hypertension in this population is critical due to consequent comorbidities and increasing numbers of bile-extracted bears finding their way to sanctuary.

## Simple Summary

Approximately 17,000 bears experience cumulative health and welfare harms in bile extraction facilities to supply traditional medicines despite the availability of proven alternatives. High prevalences of aortic dilation and renal disease were previously reported in bile-extracted bears, which are conditions both associated with systemic hypertension. We hypothesized that renal disease is positively correlated with lesions of systemic hypertension in bile-extracted bears. Archived medical records, imaging, and samples from formerly bile-extracted bears were analyzed. Hypertensive retinopathy, left ventricular hypertrophy, and aortic dilation were used as validated correlates of systemic hypertension. Our study demonstrated an unexpectedly high prevalence of systemic hypertension in this population and the most common lesions were left ventricular hypertrophy and aortic root dilation. Over 75% of bears exhibited at least one systemic hypertension lesion, and 62.8% had two or more. Lesions of systemic hypertension were positively associated with renal disease, particularly serum creatinine. Identification of additional markers of impaired renal function and systemic hypertension deserves greater investigation for earlier detection and enhanced long-term care. Understanding the etiologies contributing to systemic hypertension in this population is critical due to consequent comorbidities and increasing numbers of bile-extracted bears finding their way to sanctuary.

## 1. Introduction

Across Asia, an estimated 17,000 bears in bile extraction facilities (formerly bear bile farms) undergo repeated bile extraction to supply traditional medicinal markets and over the counter products, despite the existence of numerous proven alternatives to bear bile [1,2,3]. The most common bear species targeted, the Asiatic black bear (*Ursus thibetanus*), is listed as vulnerable by the International Union for Conservation of Nature (IUCN) [2], and under Appendix I (most endangered category) of the Convention on the International Trade of Endangered Species of Wild Fauna and Flora (CITES) [4]. Bile-extracted bears are exposed to cumulative health and welfare harms, typically confined to spatially restricted cages to facilitate bile extraction directly from the gallbladder via needle aspiration or through surgically implanted catheters or transabdominal fistulas and may undergo mutilations such as declawing or defanging [5,6,7]. Bile-extracted bears exhibit numerous concurrent behavioral and physical issues including abnormal repetitive behaviors, emaciation, dehydration, fractured teeth, bile-extraction site infections, abdominal hernias and abscesses, cholecystitis, peritonitis, neoplasia, degenerative joint disease, and missing claws, paws or limbs from being declawed and/or snare trapped in the wild [6,7,8]. Deteriorating mobility (i.e., paralysis/paresis), hepatobiliary neoplasia, and cardiovascular disease in the form of aortic aneurysm rupture/dissection are leading causes of death in previously bile-extracted bears [9]. While hepatobiliary disease and poor mobility can be directly linked to bile extraction practices and poor housing conditions in bile extraction facilities, the mechanisms leading to aortic aneurysm and rupture in this population of bears are unknown.

Left ventricular hypertrophy was previously identified in six bears that died from aortic aneurysm rupture/dissection, and a high prevalence of aortic dilation (>50%) has been reported in formerly bile-extracted Asiatic black bears, both consistent with systemic hypertension [9]. Chronic systemic hypertension contributes to comorbidities such as blindness, cerebral hemorrhage, and congestive heart failure in other species [10], prompting a critical need to understand and manage these conditions as increasing numbers of formerly bile-extracted bears with similar lesions find their way into sanctuary. Systemic hypertension is also a known risk factor for aortic dissection/rupture and has been reported in humans [11], cats [12], and gorillas [13]. Systemic hypertension is commonly attributed to underlying renal disease in other species [14,15,16], and chronic renal disease is also known to be prevalent in bile-extracted Asiatic black bears [17].

These findings led us to hypothesize that chronic renal disease is associated with subsequent systemic hypertension, left ventricular hypertrophy, and aortic dilation/aneurysm in bile-extracted Asiatic black bears. The objectives of this retrospective study were to determine if parameters of renal disease were associated with lesions of systemic hypertension in this population of bears by analyzing archived medical records and samples.

Elucidating the relationship between renal disease and lesions of systemic hypertension in bile-extracted bears will facilitate earlier recognition and treatment of bears with these conditions, and direct further investigation into etiologies and additional early-detection biomarkers of renal disease and lesions of systemic hypertension in this population of Asiatic black bears with potential applicability to other captive bears.

## 2. Materials and Methods

### 2.1. Study Population

Rescued Asiatic black bears with overt evidence of previous bile extraction (presence of implanted catheters, fistulas, and/or abdominal scarring consistent with bile extraction), cared for in a purpose-built sanctuary in Asia accredited by the Global Federation of Animal Sanctuaries (GFAS), formed the study population.

### 2.2. Data for Analysis

Medical records and samples archived between 2000 and 2018, from 251 formerly bile-extracted Asiatic black bears, both living and deceased, were reviewed. Records and samples included the following: ophthalmology examinations; thoracic radiographs; echocardiograms; serum biochemistry, urinalysis, and urine protein:creatinine (UPC) ratio results; abdominal ultrasonography; post-mortem findings and photographs; and renal tissue histopathology slides.

Due to the retrospective nature of this study, some clinical data were not available for all bears. Bears with either insufficient data to confidently categorize their systemic hypertension lesion score or deceased bears lacking archived renal histopathology samples for review were excluded, resulting in sufficient data to include 180 bears in the final analysis. Archived hematoxylin and eosin (H&E) stained renal tissue histopathology slides from 148 deceased bears were reviewed. Samples containing processing artefacts that prevented adequate interpretation of fixed tissues were excluded, resulting in samples from 81 bears included for renal histopathology scoring.

Archived (−80 °C) serum from 81 bears collected at the time of diagnosis of SHT lesions (living bears) or at death/euthanasia was available for symmetric dimethylarginine (SDMA) analysis.

Retrospective analysis of archived samples and tissues was approved by the Institution of Animal Care and Use Committee, Washington State University (#6249; 11 May 2018).

### 2.3. Sampling Procedure

Routine health checks and blood sampling, as part of bears’ long-term care in sanctuary, were performed under general anesthesia by licensed veterinarians using a combination of tiletamine–zolazepam and medetomidine for induction and isoflurane for maintenance as previously described [9,17].

Veterinary examinations have become increasingly more comprehensive since 2000 and currently consist of assessments of body condition, bodyweight, joint mobility; ophthalmic and dental examinations; abdominal ultrasonography; musculoskeletal and thoracic radiography; echocardiography; blood sampling for serum biochemistry and hematology; cystocentesis for urinalysis and, if indicated, urine protein:creatinine (UPC) ratio and/or culture and bronchoalveolar lavage (BAL) for tuberculosis (TB) testing.

Tissue sampling for histopathology was/is routinely performed wherever possible during post-mortem examinations following the unexpected death or euthanasia of bears. Euthanasia of bears in sanctuary was/is performed to alleviate suffering when quality of life or prognosis was/is deemed poor. Post-mortem kidney samples were fixed in 10% neutral buffered formalin, processed into paraffin blocks, cut into 5 µm sections and stained with hematoxylin and eosin (H&E). Due to CITES Appendix I listing of Asiatic black bears, export of tissue samples was restricted so whole slide scanning (Leica Aperio AT2, Advanced Cell Diagnostics, Beijing 100176, China) was performed to enable histopathological review of digitized light microscopy images in the United States.

### 2.4. Age Estimations

Bear age was determined by cementum annuli analysis of archived pre-molar teeth, as previously described [17,18]. Age estimates were available for 164 bears in this study.

### 2.5. Lesions of Systemic Hypertension

**Figure 1:**
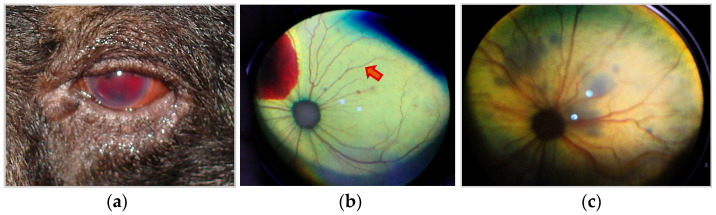
Hypertensive retinopathies identified in bile-extracted Asiatic black bears (*Ursus thibetanus*). (**a**) Hyphema/vitreal hemorrhage. (**b**) Large retinal hemorrhage and beading (red arrow) of retinal vessels. (**c**) Perivascular chorioretinal exudates. Image credits: CH.

**Figure 2:**
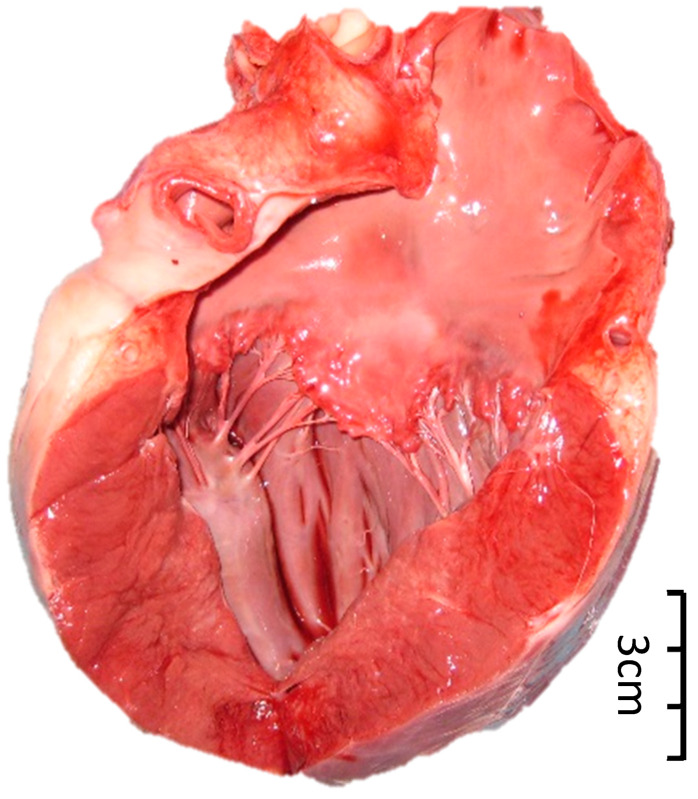
Left ventricular hypertrophy identified on post-mortem in a bile-extracted Asiatic black bear (*Ursus thibetanus*).

**Figure 3:**
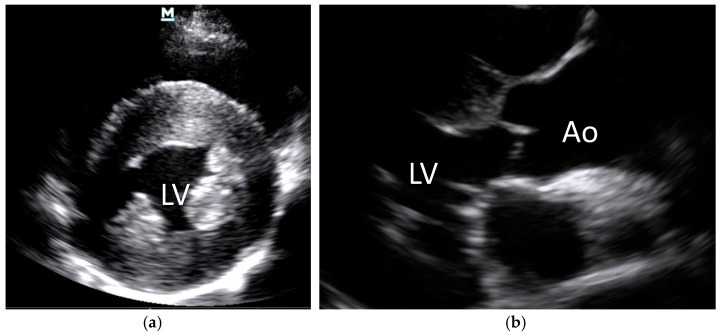
M-mode echocardiographic still frames of (**a**) left ventricular hypertrophy (short-axis view); (**b**) aortic dilation (long-axis view) in bile-extracted Asiatic black bears (*Ursus thibetanus*). LV = left ventricle; Ao = aorta.

**Figure 4:**
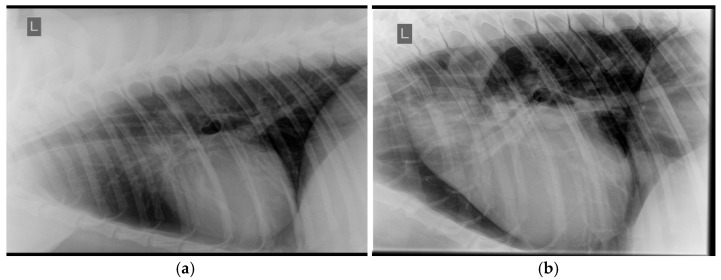
Left lateral thoracic radiographs from formerly bile-extracted Asiatic black bears (*Ursus thibetanus*): (**a**) normal aortic outflow tract and descending aorta, (**b**) severely dilated aortic outflow tract and tortuous descending aorta.

Since direct and/or conscious measurements of systemic blood pressure were not available for rescued Asiatic black bears at the time of the study, systemic hypertension (SHT) was inferred by identifying lesions indicative of end-organ damage secondary to sustained systemic hypertension validated in other species. These included hypertensive retinopathy (i.e., retinal/vitreal hemorrhage and/or retinal vessel tortuosity and/or caliber changes (beading or aneurysms) and/or perivascular chorioretinal exudates) (Figure 1), left ventricular hypertrophy (Figure 2 and Figure 3), and aortic dilation (Figure 3 and Figure 4) [14,15,19,20].

Fundus examinations were undertaken following dilation with 1% tropicamide (Mydriacyl™, Alcon Eye Care UK Ltd., Frimley, Surrey, UK) by both indirect (Keeler Vantage LED ™ indirect ophthalmoscope, Windsor, UK) and direct (Keeler professional™ direct ophthalmoscope, Windsor, UK) ophthalmoscopy by board-certified veterinary ophthalmologists (CH, DD) and trained sanctuary veterinarians. Ophthalmoscopic signs consistent with hypertensive retinopathy were confirmed by board-certified veterinary ophthalmologists (CH, DD) after the exclusion of inflammatory conditions and following a positive response to antihypertensive medications.

Echocardiography (Mindray M7 Ultrasound, Shenzen, China; P4-2s phased array 2–4 MHz transducer) was performed by veterinary cardiologists and trained sanctuary veterinarians, using guidelines adapted for animals from the American Society of Echocardiography to assess for left ventricular hypertrophy and aortic root dilation [21,22]. Echocardiographic images were measured from M-mode captured from the short axis plane of the left ventricular chamber, as is standard for veterinary echocardiography. Left ventricular measures were assessed by a board-certified veterinary cardiologist (OLN).

Left ventricular hypertrophy in deceased bears was determined from either/both pre-mortem echocardiography and post-mortem photographs of the left ventricle alongside a measuring tool. In post-mortem cases, the left ventricular free wall was measured dorsal to the papillary muscle.

Aortic dilation was identified by thoracic radiography, echocardiography (OLN, MKHB), and/or post-mortem images or records. Thoracic radiographs (left and/or right lateral and ventro-dorsal views) obtained between 2009 and 2018 were reviewed by a board-certified veterinary cardiologist (OLN), and degree of widening of the aortic outflow tract (AOT) was scored (normal, mild, moderate, or severe) as previously described [9].

### 2.6. Renal Disease

Creatinine was routinely included in serum biochemistry panels performed during health checks. Prior to 2009, blood samples were sent to a local human hospital for serum biochemical analysis, whereas samples collected after 2009 were analyzed on-site with the Idexx VetTest Chemistry Analyzer (Idexx Laboratories, Inc., Westbrook, ME 04092, USA), as previously described [17]. For this study, creatinine values recorded at the time of diagnosis of lesions of SHT (living bears) or within the preceding five months of or at death/euthanasia were used for analysis. The serum creatinine concentration reference interval cited in this study (80–219 µmol/L) was established for Asiatic black bears by Species360 Zoological Information Management System (ZIMS) [23].

Serum symmetric dimethylarginine (SDMA) concentration was obtained using the Idexx Catalyst One Chemistry Analyzer (Idexx Laboratories, Inc. Westbrook, ME 04092, USA). Samples collected at the time of diagnosis of lesions of SHT (living bears) or at death/euthanasia were included in the analysis. The reference interval for serum SDMA concentrations for healthy dogs (<14 µg/dL) was used for this study [24].

Based on preliminary analysis of digitized renal tissue images from previously bile-extracted bears, a modified scoring system based on the World Small Animal Veterinary Association Renal Pathology Initiative: Classification of Glomerular Diseases in Dogs [25] was developed for evaluating glomerular, interstitial, tubular, and vascular lesions (KT). Digital slides were evaluated in entirety, and renal histopathology lesions were scored by, or under the supervision of, a board-certified veterinary anatomic pathologist (KT) and a trained PhD candidate (MHKB); scorers were ‘blinded’ to SHT category. A subset of histopathological lesions listed by the WSAVA and appreciable by H&E-stained tissue with light microscopy alone were each scored from 0 to 4 based on a combined score incorporating frequency and severity (0 = absent, 1 = rare, 2 = mild, 3 = moderate, 4 = severe). Some features listed by WSAVA that were considered overlapping were combined. Histopathological lesions identified in these samples are listed in Table 1. The sum of lesion scores constituted the total score for each tissue sample. When multiple tissue samples were available for an individual bear, the highest score was used for statistical analysis. When multiple tissue samples were available for an individual bear, the highest score was used for statistical analysis.

### 2.7. Statistical Analysis

To test whether the presence of lesions of systemic hypertension was correlated with markers of renal disease (serum creatinine, serum SDMA, and renal histopathology), each bear was assigned to a dichotomous systemic hypertension lesion category of 0 for absence (SHT-0) or 1 for presence (SHT-1) of one or more lesions of SHT. Age (*n* = 164), serum creatinine (*n* = 164), and serum SDMA (*n* = 81) values at the date of SHT designation (living bears) or at date of death (deceased bears) and post-mortem renal histopathology score (*n* = 81) were compiled for inclusion in statistical analyses.

Sex, age, serum creatinine, serum SDMA, and renal histopathology scores were compared between the SHT-0 and SHT-1 bear groups. A Shapiro–Wilk test was performed and provided evidence against normality for all variables (*p*-value ≤ 0.05) except age (*p*-value = 0.4707). Therefore, the non-parametric Wilcoxon rank sum test was used on unimputed data for all variables except age, for which a Welch Two Sample t-test was used, to compare SHT-0 and SHT-1 groups.

Differences between SHT-0 and SHT-1 bear groups found from initial pair-wise comparisons were then further explored using logistic regression. Logistic regression with multiple imputation was performed simultaneously for all bears (live/dead), whereby the variables (age, creatinine, SDMA, and renal histopathology score) were used to impute missing data for age, creatinine, SDMA, and renal histopathology score. A logistic regression model was fit for each imputed dataset, and the multiple fits were summarized with average coefficient estimates, standard errors, and likelihoods. Likelihood ratio tests were conducted by comparing average likelihoods for full and reduced models. A *p*-value of <0.05 was considered significant.

R, Version 3.5.0 (R Core Team 2018) [26], was used for all statistical analysis; the *mice* package was used to perform multiple imputation [27], and the *rms* package was used to perform logistic regression and model averaging [28].

## 3. Results

### 3.1. Demographics

The demographic data from 180 previously bile-extracted Asiatic black bears included in this study are presented in Table 2.

### 3.2. Systemic Hypertension Lesions

The majority of the bile-extracted bears, 76.1% (137/180), exhibited at least one lesion of systemic hypertension, and 47.8% (86/180) exhibited two or more concurrent lesions. Left ventricular hypertrophy was the most common lesion (Figure 2 and Figure 3), present in 56.7% (102/180) of the bears, followed by aortic dilation/aneurysm (Figure 3 and Figure 4) in 52.8% (95/180) of the bears. Retinal hemorrhage (Figure 1) was the most common sign of hypertensive retinopathy noted by fundoscopic examination in 27.2% (49/180) of the bears, while perivascular chorioretinal exudates were identified in 13.9% (25/180) of the bears. The age and proportion of females:males were similar between the SHT-0 and SHT-1 groups, suggesting that age and sex were not factors for the development of lesions of systemic hypertension (Table 3).

### 3.3. Renal Disease Parameters

The median renal histopathology score and serum creatinine level were higher (*p* = 0.0003 and *p* = 0.0168, respectively) for the SHT-1 group compared to the SHT-0 group (Table 3).

Frequencies of the most common renal histopathology lesions identified and scored in 81 deceased bile-extracted bears are presented in Table 4. The mean age at death was not different between the SHT-0 and SHT-1 groups.

### 3.4. Associations Between Renal Disease and Lesions of Systemic Hypertension

To further explore the significant difference in renal histopathology score between the SHT-0 and SHT-1 bear groups found from the initial pair-wise comparisons above (Table 2), logistic regression was used to test whether renal histopathology score is a significant factor in the probability of developing systemic hypertension after accounting for age, serum creatinine, and SDMA. 

Logistic regression models were started with all renal parameters (serum creatinine, SDMA, and renal histopathology score). When dropping the variable ‘renal histopathology score’ to assess the effect (or contribution) from pathology (after accounting for age, serum creatinine, and SDMA), there was no evidence (*p* = 0.110) for an association between renal histopathology alone and lesions of systemic hypertension. When all three renal parameters were dropped together (and accounted for age), we found suggestive evidence (*p* = 0.062) of an association between the three combined renal parameters (serum creatinine, SDMA, and renal histopathology score) and lesions of systemic hypertension. To determine whether an association existed when restricted to diagnostic laboratory parameters available in living bears (serum creatinine and SDMA), we found suggestive evidence of an association (*p* = 0.092) between both serum creatinine and SDMA combined when both variables were dropped from the model. To determine which variable contributed more to that association, each variable was then dropped individually, and there was no evidence of an association between serum SDMA and lesions of systemic hypertension when accounting for serum creatinine and age (*p* = 0.429). However, we found a significant association between serum creatinine and lesions of systemic hypertension when accounting for serum SDMA and age (*p* = 0.043).

### 3.5. Additional Descriptive Evidence of Renal Disease in Bile-Extracted Bears

Urine protein:creatinine (UPC) ratios were available from 74 bears, and proteinuria (UPC > 0.5 based on canine reference range) was confirmed in 59.5% (44/74) of these bears. Of the bears with elevated UPC ratios, 93.2% (41/44) were also categorized as SHT-1. In addition, 41.7% (75/180) of the bile-extracted bears were diagnosed with hydronephrosis and/or hydroureter (unilateral or bilateral) via abdominal ultrasound, and a majority (82%) of these bears were also in the SHT-1 category. As the UPC ratios were not routinely measured during health checks and we have the least amount of information about these two parameters, they were not included in the statistical analyses.

## 4. Discussion

SHT is conventionally classified as either primary (idiopathic/essential) or secondary based on the absence or presence, respectively, of an underlying disease process known to elevate blood pressure [14]. In humans, primary/idiopathic SHT is most common and is attributed to a combination of genetics, lifestyle, and/or environmental factors, many of which affect the kidney’s abilities to regulate sodium excretion and extracellular fluid volume [29,30]. In dogs and cats, secondary SHT is most common and primarily attributed to renal disease as well as various endocrine disorders, obesity, and adrenal gland tumors [10]. Similar causes were considered for this population of bears. While genetic factors cannot be ruled out, renal disease was hypothesized to be the most likely etiology due to its high prevalence and a lack of evidence of other underlying conditions.

Gold standard diagnosis of SHT is accomplished by direct, intra-arterial measures of blood pressure, which is invasive and, in veterinary patients, often requires sedation or anesthesia, whereas indirect measurements (i.e., Doppler or oscillometry) are less invasive, more commonly utilized, and validated in dogs, cats, and anesthetized brown bears [31,32]. While indirect blood pressure measurements of bears in this study were routinely obtained under general anesthesia, various drugs used for (and during) anesthesia, as well as the timing at which measures were taken during the anesthetic procedures, are known to affect blood pressure results [33,34], confounding the interpretation and diagnosis of SHT by this method in this group of bears. Obtaining conscious blood pressure measurements from bears is logistically challenging, requires operant conditioning, and at the time of the study was not available for rescued bears. Therefore, indirect correlates or lesions of SHT that have been validated in other species were utilized to assess the hypertensive status in this study population.

Fundus examination is considered an efficient and non-invasive diagnostic modality for identifying systemic hypertension, where retinal arterioles can be easily visualized and can act as indicators of similar pathology in coronary and cerebral microvasculature. It has been widely acknowledged that coronary and cerebral microvasculature shares anatomical, physiological, and embryological properties with the retinal microvasculature [35]. Clinical and epidemiological studies in humans have demonstrated that hypertension is strongly correlated with many retinal microvasculature changes identifiable through fundus examination [36,37]. In particular, grades III and IV hypertensive retinopathy (retinal hemorrhages, exudates, cotton wool spots, and microaneurysms) have been associated with global cardiovascular risk [36,37,38,39]. The eye, heart, and kidney can be targets of organ damage associated with hypertension, and fundus examination has been recommended for diagnosing and monitoring progression with treatment in both humans and animals when lesions are present [40,41,42].

Lesions of SHT were present in the bile-extracted Asiatic black bears in this study, a condition, to our knowledge, not previously recognized in bear species. These findings are congruent with a previous report suspecting underlying SHT as a likely cause of aortic aneurysm and rupture in six bile-extracted Asiatic black bears [9]. The scenario of ruptured aortic root aneurysm leading to the eventual recognition of SHT prevalence in a species is not uncommon, as has been described in gorillas and cats [12,13,43]. We also previously described a higher incidence of renal disease in bile-extracted compared to non-extracted bears based on serum creatinine values [17]. The current study provides more comprehensive investigation of renal disease in bile-extracted bears using renal histopathology findings, serum creatinine, SDMA, urine protein:creatinine ratios, and the presence of structural disease such as hydroureters and hydronephrosis. Of the variables assessed, the renal histopathology score and serum creatinine were significantly higher in bears with lesions of SHT.

Renal disease is a major cause of SHT in humans, dogs, and cats [14,15,44,45,46], and hypertension induced by chronic kidney disease was recently reported in leopards [47]. Renal disease can develop as a result of injury to renal vasculature, tubules, interstitium, or glomeruli, which can lead to an irreversible and/or a progressive loss of renal function, chronic kidney disease, and subsequent SHT [16,48,49]. Examples of inciting causes of renal injury include systemic disease, toxins, nephrotoxic drugs, infections/infectious disease, immune complex deposition, ischemia, and urinary tract obstruction [49,50,51]. We suspect that bile-extracted bears have multiple sources of chronic bacterial infections that could induce glomerular/renal disease, the most salient being bile-extraction site infections. Exposure to nephrotoxic drugs could also be a contributor to renal disease, as bears in extraction facilities are administered a range of medications, often without qualified veterinary guidance. Associations between medications administered pre-rescue and the development of renal disease are difficult to investigate, as medical records from bile extraction facilities are often non-existent [6,52].

The association between serum creatinine and lesions of SHT was greater compared to other renal parameters in bile-extracted bears. The highest elevations of serum creatinine were seen in the SHT-1 bear group. Elevations in serum creatinine do not occur until there is a loss of 75% of functioning nephrons [53], which, unfortunately, makes serum creatinine a relatively insensitive marker to detect early renal disease. This is an important problem given that bile-extracted bears have been shown to have a high incidence of chronic renal disease associated with reduced survival [17].

SDMA, a byproduct of protein methylation normally excreted by the kidneys, is a validated indirect biomarker of glomerular filtration rate that is less affected by non-renal factors such as muscle mass and is reportedly more sensitive at detecting early renal disease compared to creatinine in humans, dogs, cats, and cheetahs [53,54,55,56]. While SDMA has not been specifically validated in bears, its validation in domestic carnivores and cheetahs suggests its homology is well conserved across species and could therefore be a useful renal biomarker in bears [56]. In the bears of this study, however, we did not find a significant benefit for SDMA in any of our models. The sample size may have been too small to detect an effect of SDMA or SDMA may not be a reliable marker to assess glomerular filtration rate in bears, perhaps similar to findings that serum SDMA and GFR were poorly correlated in hyperthyroid cats [57].

Advanced age can be associated with renal pathology, particularly global glomerulosclerosis, interstitial fibrosis, and tubular atrophy in dogs and humans [58]. However, the mean age at death of bears in this study was 17 (±4.6) years, which is approximately half the expected lifespan of a healthy, captive bear, suggesting that advanced age may be less of a contributor to the pathology identified in these bears.

In humans, chronic stress has also been associated with the development of SHT [59]. We cannot discount the potential effect of chronic stress as a contributor to lesions associated with SHT in this population of bears as markers of stress or anxiety were not evaluated. Wildlife species held in inappropriate captive environments can experience multiple sources of chronic stress [60], leading to activation of the hypothalamic–pituitary–adrenal (HPA) axis and increased secretion of glucocorticoids [61]. Studies have documented elevated cortisol levels in hair samples from bears confined in inappropriate conditions prior to rehabilitation in accredited sanctuary [61], consistent with a stress response that could contribute to SHT. Examples of multiple, concurrent stressors and sources of physical and psychological trauma previously documented for this population of bears include, but are not limited to, restrictive confinement, profound behavioral deprivation, malnutrition, painful procedures, fear, anxiety, and chronic inflammation and infection [6,7,8]. The contribution of stress to the development of lesions of SHT in this population of bears deserves greater investigation.

## 5. Limitations

The limitations of blood pressure measurements in this species have been discussed. There are common limitations to retrospective studies such as missing data points in some subjects. Even so, we believe that the number of animals included in the analysis is robust. While histopathology is the gold standard for characterization and diagnosis of renal disease, there were limitations to this method such as the tissue sample size relative to the heterogeneity of disease present. It is possible that transmission electron microscopy (TEM) or additional processing and special staining of archived renal tissues could provide additional diagnostic information that may affect the interpretation of renal pathology of these samples.

## 6. Conclusions

Our study demonstrates an unexpectedly high prevalence of lesions associated with SHT in formerly bile-extracted Asiatic black bears, presenting a condition, to our knowledge, not previously recognized in bear species. The most common lesions were left ventricular hypertrophy and aortic root dilation. Lesions of SHT were positively associated with renal disease, particularly serum creatinine levels and are not the result of advanced age. SDMA does not appear to be a valuable predictor of lesions of SHT or renal disease in this population. Additional markers of impaired glomerular filtration rate, and SHT evaluations are needed to facilitate earlier detection of these conditions in bears that have had bile extraction to enhance their rehabilitation and long-term health and welfare.

## Figures and Tables

**Table 1:** Renal histopathology lesions assessed and scored from archived post-mortem tissue slides from 81 previously bile-extracted Asiatic black bears (*Ursus thibetanus*).

Glomerular Tufts/ Corpuscles	Interstitium	Vascular	Tubules
Hypercellularity	Fibrosis	Medial hypertrophy	Tubular necrosis
Neutrophils	Inflammation	Arterial hyalinosis	Single cell necrosis
Increased mesangium	Edema		Regeneration
Obsolescence	Hemorrhage		Proteinosis
Synechia			Intratubular crystals
Bowman’s space ectasia			Epithelial cell pigment
Parietal cell hypertrophy			Intraluminal pigment
Crescent/fibrin			Tubulitis
Capsular thickening			Dilation/Ectasia
Protein in Bowman’s space			

**Table 2:** Demographics (sex, age, and status) and systemic hypertension lesion category based on lesions of systemic hypertension of 180 previously bile-extracted Asiatic black bears (*Ursus thibetanus*).

Demographics	Females	Males	Total
Number of bears	115 (64%)	65 (36%)	180
Mean Age ^a^ (years) ± SD (range)	17 ± 4.7 (5–27)	18 ± 4.4 (3–28)	17 ± 4.6 (3–28)
Status			
Living Dead	67 (58.3%) 48 (41.7%)	32 (49.3%) 33 (50.7%)	99 (55%) 81 (45%)
Hypertension Category:			
SHT-0 SHT-1	28 (24.3%) 87 (75.7%)	15 (23.1%) 50 (76.9%)	43 (23.9%) 137 (76.1%)

^**a**^ Age at death or at designation of systemic hypertension (SHT) category, age estimates missing for 16 bears (6 males;10 females). SHT-0 = no lesions of systemic hypertension; SHT-1 = at least one lesion of systemic hypertension identified. SD = standard deviation.

**Table 3:** Pairwise comparison of sex, and median age, serum creatinine, SDMA, and renal histopathology score between SHT-0 and SHT-1 Asiatic black bear (*Ursus thibetanus*) groups.

	SHT-0 (*n* = 43)	SHT-1 (*n* = 137)	*p*-Value
Female	28 (65.1%)	87 (63.5%)	
Male	15 (34.9%)	50 (36.5%)	
Median Age ^a^ (yrs) (range) (*n* = 164)	13 (8–21)	11 (1–28)	0.2503 ^b^
Median Serum CREA (µmol/L) (range) (*n* = 169)	140 (59–503)	153 (53–1516)	0.0168 ^c^
Median Serum SDMA (µg/dL) (range) (*n* = 81)	12 (8–33)	12 (7–100)	0.3664 ^c^
Median Renal Histopathology Score (range) (*n* = 81)	3 (0–24)	8 (0–29)	0.0003 ^c^

^**a**^ Age at death or at designation of SHT category; ^**b**^ Welch Two Sample *t*-test; ^**c**^ Wilcoxon rank sum test; SHT-0 = no lesions of systemic hypertension; SHT-1 = one or more lesions of systemic hypertension. CREA = serum creatinine (RR 80–219 μmol/L) [23]; SDMA = serum symmetric dimethylarginine (upper limit of normal 14 μg/dL) [24].

**Table 4:** Most common renal histopathological lesions identified in archived H&E-stained renal tissue samples from 81 deceased bile-extracted Asiatic black bears comparing numbers and percentages of bears exhibiting lesions between SHT-0 and SHT-1 bear groups.

	SHT-0 (*n* = 18)	SHT-1 (*n* = 63)
**Mean Age at Death ^a^ (years) ± SD** (range)	16 ± 3.3 (9–21)	17 ± 4.8 (7–28)
**Glomerular Tuft Lesions:**	No. of bears (%)	No. of bears (%)
Obsolescence (*n* = 40)	3 (16.7%)	37 (58.7%)
Bowman’s space ectasia (*n* = 40)	6 (33.3%)	34 (54.0%)
Increased mesangium (*n* = 26)	3 (16.7%)	23 (36.5%)
Capsular thickening (*n* = 25)	1 (5.6%)	24 (38.1%)
Protein (*n* = 24)	4 (22.2%)	20 (31.7%)
Synechia (*n* = 11)	1 (5.6%)	10 (15.9%)
**Interstitial Lesions**		
Fibrosis (*n* = 59)	6 (33.3%)	53 (84.1%)
Inflammation (*n* = 36)	4 (22.2%)	32 (50.8%)
**Tubular Lesions**		
Dilation/Ectatic (*n* = 45)	6 (33.3%)	39 (61.9%)
Proteinosis (*n* = 41)	7 (38.9%)	34 (54.0%)
Regeneration (*n* = 20)	1 (5.6%)	19 (30.2%)

^**a**^ Age estimates were available for 65 of the 81 deceased bears; SHT-0 = no lesions of systemic hypertension; SHT-1 = one or more lesions of systemic hypertension; H&E = hematoxylin and eosin.

## Data Availability

Restrictions apply to the availability of these data. The data were obtained from the Animals Asia Foundation and are available from the corresponding author (M.K.H.B.) with the permission of the Animals Asia Foundation.

## Abbreviations

The following abbreviations are used in this manuscript:

CITES	Convention on the International Trade of Endangered Species of Wild Fauna and Flora
SDMA	Symmetric dimethylarginine
SHT	Systemic hypertension
SHT-0	Category of bears with no lesions of systemic hypertension
SHT-1	Category of bears with one or more lesions of systemic hypertension
UPC	Urine protein:creatinine ratio
